# Prolific Production

**Published:** 1890-08

**Authors:** 


### PROLIFIC PRODUCTION.

According to naturalists a scorpion will produce 65 young; a common fly will lay 144 eggs, a leach 150, and a spider 170. A hydrachina produces 600 eggs, and a frog 1,100. A female moth will produce 1,100 eggs, and a tortoise 1,000. A gall insect has laid 50,000 eggs; a shrimp 6,000; and 10,000 have been found in the ovary of an ascaris.

One naturalist found over 12,000 eggs in a lobster, and another over 21,000. An insect very similar to an ant (*Mutilla*) has produced 80,000 eggs in a single day; and Leuwenhoeck seems to compute 4,000,000 to the crab's share.

Many fishes produce an incredible number of eggs. More than 36,000 have been counted in a herring; 38,000 in a smelt; 1,000,000 in a sole; 1,130,000 in a roach; 3,000,000 in a sturgeon; 342,000 in a carp; 383,000 in a tench; 546,000 in a mackerel; 992,000 in a perch, 1,357,000 in a flounder. But of all of the fishes hitherto discovered, the cod seems to be the most prolific.

One naturalist computes that this fish produces more than 3,686,000 eggs, and another as many as 9,444,000. A rough calculation has shown that, were 1 per cent. of the eggs of the salmon to result in full grown fish, and were they and their progeny to continue to increase in the same ratio, they would, in about sixty years, amount in bulk to many times the size of the earth. Nor is the salmon the most prolific of species. In a yellow perch weighing 3 1-2 ounces have been counted 9,943 eggs, and in a smelt ten inches and a half in length, 25,141.

An interesting experiment was made in Sweden in 1761, by Charles F. Lund. He obtained from 50 female breams 3,100,000 young; from 100 perch, 3,125,000 young; and from 100 female mullets, 4,000,000 young.

A 6 1-2 inch Sunfish (weighing 5 1-2 ounces) has been estimated as carrying 44,000 eggs. The female deposits them in a shallow, sandy place, and the male guards the nest.

